# Regulation of Seasonal Reproduction by Hypothalamic Activation of Thyroid Hormone

**DOI:** 10.3389/fendo.2014.00012

**Published:** 2014-02-21

**Authors:** Ai Shinomiya, Tsuyoshi Shimmura, Taeko Nishiwaki-Ohkawa, Takashi Yoshimura

**Affiliations:** ^1^Division of Seasonal Biology, National Institute for Basic Biology, Okazaki, Japan; ^2^Laboratory of Animal Physiology, Graduate School of Bioagricultural Sciences, Nagoya University, Nagoya, Japan; ^3^Institute of Transformative Bio-Molecules (WPI-ITbM), Nagoya University, Nagoya, Japan; ^4^Avian Bioscience Research Center, Graduate School of Bioagricultural Sciences, Nagoya University, Nagoya, Japan

**Keywords:** seasonal reproduction, mediobasal hypothalamus, ependymal cell, pars tuberalis, thyrotropin, thyroid hormone, iodothyronine deiodinase

## Abstract

Organisms living outside the tropics measure the changes in the length of the day to adapt to seasonal changes in the environment. Animals that breed during spring and summer are called long-day breeders, while those that breed during fall are called short-day breeders. Although the influence of thyroid hormone in the regulation of seasonal reproduction has been known for several decades, its precise mechanism remained unknown. Recent studies revealed that the activation of thyroid hormone within the mediobasal hypothalamus plays a key role in this phenomenon. This localized activation of the thyroid hormone is controlled by thyrotropin (thyroid-stimulating hormone) secreted from the pars tuberalis of the pituitary gland. Although seasonal reproduction is a rate-limiting factor in animal production, genes involved in photoperiodic signal transduction pathway could emerge as potential targets to facilitate domestication.

## Introduction

**Figure 1:**
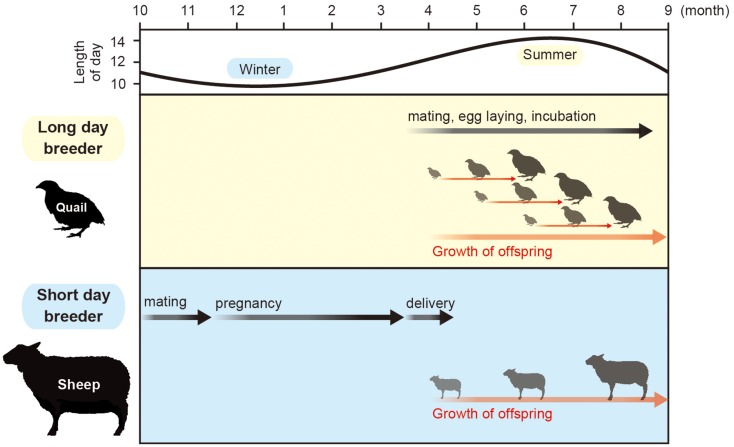
**Calendar of seasonal breeding animals**. Most animals mate in a specific time of a year. Small animals with short gestation or incubation period mate in spring and summer, while large animals that have a 6-month gestation period mate in fall to give birth in spring.

Orbiting of the earth around the sun causes changing seasons. To adapt to the seasonal changes in the environment, animals alter their physiology and behavior, which is characterized by the changes in growth, metabolism, immune function, reproductive activity, migration, hibernation, and molting. Most of the organisms use the changes in the length of the day (photoperiod) as a calendar, because temperature and precipitation varies throughout each year and are unreliable when compared with the length of the day. This phenomenon is called “photoperiodism” (1). Among the various seasonally regulated phenomena, the mechanism of seasonal reproduction has been extensively studied. Small mammals and birds breed during the spring and summer. Therefore, they are called long-day (LD) breeders. The gestation or incubation period of these animals last only a few weeks and their offspring are born during the spring and summer. In contrast, larger mammals, such as goats and sheep, breed during fall. Therefore, they are called short-day (SD) breeders. These animals have a gestation period of approximately 6 months. Therefore, their offspring are also born and raised during spring and summer. Accordingly, the offspring of both LD and SD breeders grow when the climate is moderate and food is abundant (Figure 1).

Seasonal reproduction of vertebrate species is regulated by the hypothalamic–pituitary–gonadal (HPG) axis. The secretion of gonadotropin-releasing hormone (GnRH) from the hypothalamus induces the secretion of gonadotropins [luteinizing hormone (LH) and follicle-stimulating hormone (FSH)] from the anterior pituitary gland, which in turn activates gonadal activity. In other words, the HPG axis of seasonally breeding animals is only activated during the breeding season. Among the various vertebrate species, birds show the most dramatic changes in gonadal size (typically more than a 100-fold) (2). Therefore, birds have a highly sophisticated photoperiodic mechanism in comparison to other vertebrate species (3). In addition to the robust gonadal responses, most of the birds have very short breeding seasons, as the HPG axis is automatically switched off and their gonads start to regress even though the length of the day is still increasing. This phenomenon is known as photorefractoriness (4, 5). The length of the breeding season tends to be shorter in higher latitude due to the short benign season in higher latitude. Among mammals, hamsters and sheep are extensively studied, because they show dramatic photoperiodic responses. However, the magnitude of the seasonal gonadal development and regression is less robust in mammals than in birds, as their gonads change only by a few-folds.

## Influence of Thyroid Hormone in the Seasonal Changes

It has been known for many decades that thyroid hormone is somehow involved in the regulation of seasonal reproductive function in various organisms including fish, birds, and mammals (2, 6, 7). In some species, thyroidectomy prevents the transition to reproductive state (i.e., seasonal testicular development and/or regression) (8–11), and thyroxine (T_4_) treatment mimics the effects of a long photoperiod (12–14). However, photo-stimulated gonadal maturation appears to have been largely unaffected by thyroidectomy in some species (2). Therefore, the reported effects of thyroidectomy on seasonal breeding are often contradictory and the role of T_4_ is thought to be permissive. Although the requirement of T_4_ for an appropriate response to photoperiod has been documented (15), the mechanism by which thyroid hormone regulates seasonal reproduction remained unknown for several decades.

## Photoperiodic Changes in Type 2 and Type 3 Deiodinases Within the Hypothalamus

**Figure 2:**
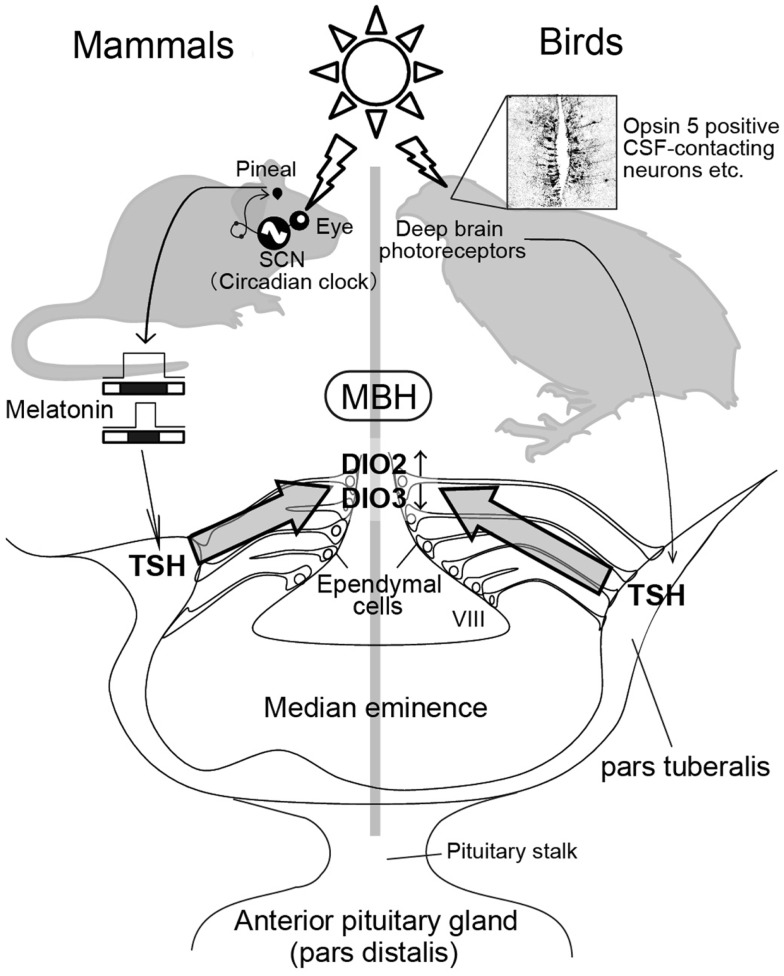
**Photoperiodic signal transduction pathway in mammals and birds**. In mammals, light information is received by the eye and transmitted to the pineal gland via the circadian pacemaker, the suprachiasmatic nucleus (SCN). The duration of the pineal melatonin signal encodes the length of night and regulates TSH secretion in the pars tuberalis. The pars tuberalis TSH acts on TSH receptor expressed in the ependymal cells lining ventrolateral walls of the third ventricle (VIII) to induce DIO2 and reduce DIO3. Local thyroid hormone activation within the mediobasal hypothalamus (MBH) by DIO2/DIO3 switching plays a key role in the regulation of seasonal reproduction. In contrast, light information received by deep brain photoreceptors induces TSH secretion from the pars tuberalis in birds. Nevertheless, melatonin is not involved in the seasonal reproduction of birds. The schematic is a modified version of illustration published by Ikegami and Yoshimura (40).

The Japanese quail (*Coturnix japonica*) is an excellent model for studying photoperiodism, because of its rapid and robust responses to changing photoperiods (3). Local illumination of the mediobasal hypothalamus (MBH) by radioluminous-painted beads induce testicular growth (16), and lesions of MBH blocks the photoperiodic response of LH secretion and gonadal development (17, 18). In addition, expression of c-Fos, a marker of neuronal activation, is induced in the MBH by LD stimulus (19). The MBH is therefore considered central for the seasonal reproduction in quail. By using differential subtractive hybridization analysis, LD-induction of type 2 deiodinase gene (*DIO2*) and LD-suppression of type 3 deiodinase gene (*DIO3*) were observed in the ependymal cells (also known as tanycytes) that line the ventrolateral walls of the third ventricle within the MBH [Ref. (20, 21), Figure 2]. *DIO2* encodes the thyroid hormone-activating enzyme that converts the prohormone T_4_ to bioactive triiodothyronine (T_3_) (22), while *DIO3* encodes thyroid hormone-inactivating enzyme that metabolizes T_4_ and T_3_ to inactive reverse T_3_ (rT_3_) and 3,3′-diiodothyronine (T_2_), respectively. The reciprocal switching of *DIO2* and *DIO3* appears to regulate the local thyroid hormone concentration precisely within the MBH. Moreover, T_3_ concentration within the MBH is about 10-fold higher under LD conditions than under SD conditions, even though plasma concentrations are similar to both photoperiods (20). The functional significance of this locally activated thyroid hormone has been demonstrated by pharmacological analyses. Intracerebroventricular (i.c.v.) infusion of T_3_ in SD conditions induced testicular development while infusion of a DIO2 inhibitor (iopanoic acid) in LD conditions attenuated testicular development (20). Photoperiodic regulation of *DIO2* and/or *DIO3* has also been confirmed in a number of other avian species, such as the tree sparrow (23), chicken (24), great tits (25), and canary (26). Similarly, photoperiodic regulation of thyroid hormone metabolism in the MBH has been confirmed in various mammalian species, including LD breeders like Siberian hamsters (27–30), Syrian hamsters (31, 32), rats (33, 34), mice (35), and SD-breeding goats (36) and sheep (37). Activation of thyroid hormone within the MBH decodes the LD information. Therefore, daily T_3_ subcutaneous injections induce testicular development (28) and chronic replacement of T_3_ in the hypothalamus prevents the onset of testicular regression (27) in LD-breeding Siberian hamsters. In contrast, in the SD breeders, LD-induced DIO2 appears to convert T_4_ to T_3_ to terminate the breeding season (37). In addition, LD stimulus induces the expression of *DIO2*, and T_4_ administration terminates the breeding season via a decrease in serum LH (38, 39).

## Thyroid Hormone Transport to the Ependymal Cells

Due to their lipophilic nature, thyroid hormones are believed to traverse plasma membranes by passive diffusion. However, involvement of a membrane transport system for thyroid hormone has been reported recently and a mechanism that facilitates the transport of thyroid hormone into the ependymal cells was examined. Some members of the organic anion transporting polypeptide (Oatp) family have been shown to transport thyroid hormones in mammals (41, 42) and the involvement of a member of this family in transporting T_4_ into the quail brain has been investigated (43). Oatp1c1, which is expressed in the ependymal cells within the MBH, has been demonstrated to be a highly specific transporter of T_4_. In addition to Oatp1c1, another thyroid hormone transporter, monocarboxylate transporter 8 (MCT8), has been found in the ependymal cells within hamster MBH (29). Although MCT8 appears to be involved in the regulation of photoperiodism, its expression is upregulated under SD conditions, which does not require thyroid hormone.

## Regulation of Hypothalamic Deiodinases by the Pars Tuberalis TSH

When quail are transferred from SD conditions to LD conditions, an increase in plasma gonadotropin (LH) is observed 22 h after the dawn of the first LD (3, 44, 45). As discussed previously, reciprocal switching of *DIO2* and *DIO3* plays a critical role in the regulation of seasonal reproduction in birds and mammals. In quail, the reciprocal switching of *DIO2* and *DIO3* precedes photoperiodic induction of gonadotropin release by roughly 4 h (21). Genome-wide gene expression analysis during the transition from SD conditions to LD conditions in Japanese quail (45) identified the induction of two genes 4 h prior to *DIO2/DIO*3 switching (i.e., 14 h after dawn) in the pars tuberalis of the pituitary gland. The pars tuberalis consists of thin layers of cells surrounding the median eminence (Figure 2). One of these genes encode the thyroid-stimulating hormone β subunit (*TSHB*) and the other encode the transcriptional co-activator eyes absent 3 (*EYA3*). Although EYA3 is a transcriptional co-activator, the expression sites of *EYA3* and *DIO2*/*DIO3* are different (i.e., *EYA3* in the pars tuberalis and *DIO2/DIO3* in the ependymal cells). Therefore, it appears that EYA3 is not involved in the regulation of *DIO2*/*DIO3* switching. On the other hand, the expression of TSH receptor (TSHR) and binding of ^125^I-labeled thyroid-stimulating hormone (TSH) were observed in the ependymal cells where *DIO2* and *DIO3* are expressed. In addition to these, i.c.v. TSH administration induced *DIO2* expression and reduced *DIO3* expression in the ependymal cells even under SD conditions, while passive immunization against TSH attenuated LD-induction of *DIO2* expression (45). The involvement of TSHR-Gsα-cAMP signaling pathway in this TSH regulation of *DIO2* expression was demonstrated by the promoter analysis. Considering that the magnitude of testicular growth induced by i.c.v. TSH infusion was almost similar to that observed in birds exposed to LD stimulus, the LD-induced pars tuberalis TSH appears to be a major factor regulating the seasonal reproduction in birds.

In birds, eyes are not necessary for the regulation of seasonal reproduction because deep brain photoreceptors are involved in this process (46, 47). Although pineal organ is a photoreceptive organ in non-mammalian vertebrates (48, 49), pineal organ is not involved in the regulation of seasonal reproduction (50, 51). In contrast, local illumination of the septal region of the telencephalon or the MBH using radioluminous-painted beads caused testicular growth in quail, suggesting the existence of deep brain photoreceptors in these regions (16). Localization of several rhodopsin family proteins (rhodopsin; OPN4: melanopsin; OPN5: neuropsin and VA opsin: vertebrate ancient opsin) are reported in these brain regions and projections that link some of these photoreceptor cells to the pars tuberalis have also been reported (52–62). These photoreceptors are therefore thought to be involved in the seasonal regulation of reproduction in birds (Figure 2).

In a marked contrast to avian species, eyes are the only photoreceptive organ in mammalian species (63–69). Therefore, removal of the eyes abolishes the photoperiodic response (64, 68). Light information received by the eye is transmitted to the pineal gland through the suprachiasmatic nucleus (SCN), where the circadian pacemaker is localized (68, 70–74). The duration of night corresponds to the nocturnal secretion profile of melatonin, which plays a crucial role in the regulation of seasonal reproduction in mammalian species. For example, in both LD and SD breeders, pinealectomy abolishes seasonal responses, while melatonin administration restores them (68, 74, 75). Melatonin acts via melatonin receptors and there are two subtypes of melatonin receptors (MT1 and MT2) in mammals (76, 77). However, these melatonin receptors are not expressed in the ependymal cells where *DIO2* and *DIO*3 are expressed (78, 79). The MT1 receptor is reportedly expressed in the thyrotroph cells of the pars tuberalis (80, 81). Therefore, pars tuberalis TSH likely mediates the influence of melatonin in the *DIO2*/*DIO3* switching in mammalian species. Although it is generally considered that laboratory mice are non-seasonal breeders, many researchers noticed that mice do not breed well during the winter (e.g., small litter size) even though they are kept under standardized conditions. To determine whether pars tuberalis TSH mediates the influence of melatonin in the *DIO2/DIO3* switching, laboratory mice were analyzed as experimental models. Two key enzymes, arylalkylamine *N*-acetyltransferase (AA-NAT) and hydroxyindole-*O*-methyltransferase (HIOMT) are involved in melatonin biosynthesis from serotonin (74). However, most inbred mice genetically lack the ability to produce these enzymes, resulting in minimal melatonin generation (82, 83). Therefore, it was predicted that melatonin-producing strains would have the capacity to respond to photoperiodic changes, while melatonin-deficient strains would be resilient to such changes. As expected, clear photoperiodic regulation of *TSHB, DIO2*, and *DIO3* was observed in the melatonin-producing CBA strain, while such responses were not observed in the melatonin-deficient C57BL strain (35). In addition, daily intraperitoneal (i.p.) melatonin injections mimicked the effect of SD conditions on the expression of these genes (35). To test the involvement of the TSH–TSHR signaling pathway in the melatonin-mediated regulation of *DIO2*/*DIO3* expression, the effects of melatonin administration were examined in TSHR-null mice (35). The TSHR-null mice failed to respond to melatonin administration. This result clearly suggested the involvement of a TSH–TSHR signaling pathway in the melatonin-mediated regulation of *DIO2*/*DIO3* in mammals. In addition, the analysis of mice that lacked the MT1 and MT2 melatonin receptors revealed the involvement of MT1 melatonin receptors in this regulation (84). It is also interesting to note that TSH is involved in the LD-induction of *DIO2* in SD-breeding sheep (37). Thus, pars tuberalis TSH appears to relay the seasonal information in both LD and SD-breeding animals and sensitize them for spring.

## Thyroid Hormone Action within the Hypothalamus

**Figure 3:**
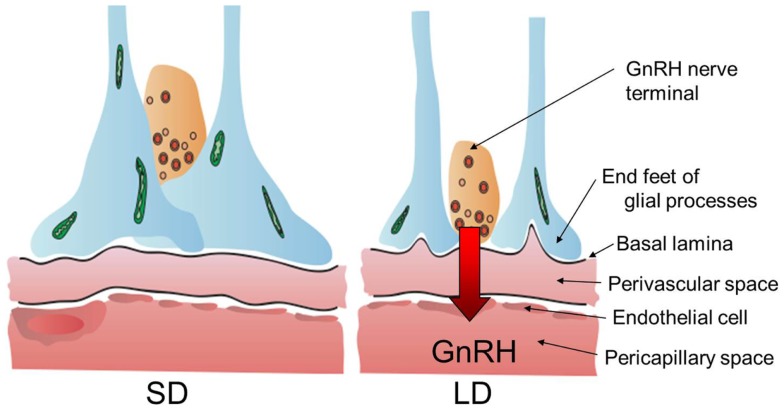
**Neuro–glial interaction between GnRH nerve terminals and glial endfeet**. Locally activated thyroid hormone within the MBH regulates neuro–glial interaction in the median eminence and these morphological changes appear to regulate or modulate seasonal GnRH secretion from the hypothalamus to portal capillary. The illustration has been modified from that published by Yoshimura (89).

Thyroid hormone is involved in the development and plasticity of the central nervous system (22). The expression of thyroid hormone receptors (*THR*α, *THR*β, and *RXR*α) in the median eminence suggested that the median eminence is the target site of action for the photo-induced increase in T_3_ in the quail MBH (20). To understand the action of thyroid hormone within the MBH, the ultrastructure of the median eminence was examined under SD and LD conditions using electron microscopy. Dynamic morphological changes were observed between the GnRH nerve terminals and glial endfeet within the median eminence (85). In SD conditions, many GnRH nerve terminals are encased by the endfeet of glial processes and do not contact the basal lamina, while many GnRH nerve terminals are in close proximity to the basal lamina under LD conditions (Figure 3). It has been proposed that the nerve terminals of hypothalamic neurons are required to directly contact the pericapillary space for the secretion of the hypothalamic neurohormone from the hypothalamus into the portal capillary (86). Morphological changes between the GnRH nerve terminals and endfeet of glial processes are observed in SD quail treated with T_3_ to stimulate testicular growth (87). Therefore, these morphological changes appear to regulate or modulate the seasonal GnRH secretion from the median eminence. It is also interesting to note that the seasonal plasticity within the GnRH system is reported in ewes (88).

## Photoperiodic Signaling Pathway and Domestication

Seasonal reproduction is a rate-limiting factor for the animal procreation. The photoperiodic signaling pathway could also be a potential target that facilitates human-driven domestication process. As discussed previously, most laboratory mice lack the enzyme activity of melatonin biosynthesis pathway (82, 83, 90, 91). In addition, occurrence of selective sweeps was found at the TSHR locus in all domestic chickens (92). This observation suggests that the TSHR may be a domestication locus in chicken (92). Although we still do not know the correlation with domestication, it is interesting to note that photoperiodic regulation of *DIO3* is absent in Syrian hamster (27). Thus, genes involved in the photoperiodic signaling pathway could emerge as useful targets for the domestication of wild animals.

## Conclusion

Involvement of thyroid hormone in the regulation of seasonal reproduction has been suggested in the past several decades. Recent comparative studies clearly reveal that the local activation of thyroid hormone within the hypothalamus is a key factor in the regulation of seasonal reproduction in a number of mammalian and avian species. It is important to note that this mechanism is also conserved in fish (93) and is universal among various vertebrate species. Although thyroid hormone influences both LD and SD breeders, the mechanism that differentiates LD breeders from SD breeders remains unknown. Presumably, the responsiveness of pathways downstream of T_3_ activity (e.g., responsiveness of T_3_ target genes to LD-induced T_3_ etc.) differs in LD and SD breeders. The switching mechanism of LD breeder and SD breeder needs to be clarified in the future studies.

## Conflict of Interest Statement

The authors declare that the research was conducted in the absence of any commercial or financial relationships that could be construed as a potential conflict of interest.

## References

[1] Garner WW Allard HA Effect of the relative length of day and night and other factors of the environment on growth and reproduction in plants. J Agric Res (1920) 18:553–606

[2] Dawson A King VM Bentley GE Ball GF Photoperiodic control of seasonality in birds. J Biol Rhythms (2001) 16:365–80 10.1177/074873001129002079 11506381

[3] Follett BK King VM Meddle SL Rhythms and photoperiodism in birds. In: Lumsden PJ Miller AJ editors. Biological Rhythms and Photoperiodism in Plants. Oxford: Biostatistics Scientific (1998). p. 231–42

[4] Hahn TP MacDougall-Shackleton SA Adaptive specialization, conditional plasticity and phylogenetic history in the reproductive cue response systems of birds. Philos Trans R Soc Lond B Biol Sci (2008) 363:267–86 10.1098/rstb.2007.2139 17686737 PMC2606750

[5] Nicholls TJ Goldsmith AR Dawson A Photorefractoriness in birds and comparison with mammals. Physiol Rev (1988) 68:133–76 3275941 10.1152/physrev.1988.68.1.133

[6] Cyr DG Eales JG Interrelationships between thyroidal and reproductive endocrine systems in fish. Rev Fish Biol Fish (1996) 6:165–200 10.1007/BF00182342

[7] Nicholls TJ Follett BK Goldsmith AR Pearson H Possible homologies between photorefractoriness in sheep and birds: the effect of thyroidectomy on the length of the ewe’s breeding season. Reprod Nutr Dev (1988) 28:375–85 10.1051/rnd:19880304 3413338

[8] Moenter SM Woodfill CJ Karsch FJ Role of the thyroid gland in seasonal reproduction: thyroidectomy blocks seasonal suppression of reproductive neuroendocrine activity in ewes. Endocrinology (1991) 128:1337–44 10.1210/endo-128-3-1337 1999155

[9] Dawson A Thyroidectomy progressively renders the reproductive system of starlings (*Sturnus vulgaris*) unresponsive to changes in daylength. J Endocrinol (1993) 139:51–5 10.1677/joe.0.1390051 8254293

[10] Dawson A Thyroidectomy of house sparrows (*Passer domesticus*) prevents photo-induced testicular growth but not the increased hypothalamic gonadotrophin-releasing hormone. Gen Comp Endocrinol (1998) 110:196–200 10.1006/gcen.1998.7065 9570940

[11] Parkinson TJ Follett BK Thyroidectomy abolishes seasonal testicular cycles of Soay rams. Proc Biol Sci (1995) 259:1–6 10.1098/rspb.1995.0001 7700874

[12] Follett BK Nicholls TJ Influences of thyroidectomy and thyroxine replacement on photoperiodically controlled reproduction in quail. J Endocrinol (1985) 107:211–21 10.1677/joe.0.1070211 4067480

[13] Goldsmiths AR Nicholls TJ Thyroxine effects upon reproduction, prolactin secretion and plumage moult in thyroidectomised European starlings *Sturnus vulgaris*. Ornis Scand (1992) 23:398–404 10.2307/3676666

[14] Wilson FE Reinert BD Thyroid hormone acts centrally to programme photostimulated male American tree sparrows (*Spizella arborea*) for vernal and autumnal components of seasonality. J Neuroendocrinol (2000) 12:87–95 10.1046/j.1365-2826.2000.00437.x 10692147

[15] Bentley GE Photoperiodism and reproduction in birds. In: Nelson RJ Denlinger DL Somers DE editors. Photoperiodism: The Biological Calendar. New York: Oxford University Press (2010). p. 420–45

[16] Homma K Ohta M Sakakibara Y Photoinducible phase of the Japanese quail detected by direct stimulation of the brain. In: Suda M Hayaishi O Nakagawa H editors. Biological Rhythms and Their Central Mechanism. Amsterdam: Elsevier (1979). p. 85–94

[17] Sharp PJ Follett BK The effect of hypothalamic lesions on gonadotrophin release in Japanese quail (*Coturnix coturnix japonica*). Neuroendocrinology (1969) 5:205–18 10.1159/000121861 5362399

[18] Juss TS Meddle SL Servant RS King VM Melatonin and photoperiodic time measurement in Japanese quail (*Coturnix coturnix japonica*). Proc R Soc Lond B Biol Sci (1993) 254:21–8 10.1098/rspb.1993.0121 8265672

[19] Meddle SL Follett BK Photoperiodically driven changes in Fos expression within the basal tuberal hypothalamus and median eminence of Japanese quail. J Neuroscience (1997) 17:8909–18 9348357 10.1523/JNEUROSCI.17-22-08909.1997 PMC6573072

[20] Yoshimura T Yasuo S Watanabe M Iigo M Yamamura T Hirunagi K Light-induced hormone conversion of T_4_ to T_3_ regulates photoperiodic response of gonads in birds. Nature (2003) 426:178–81 10.1038/nature02117 14614506

[21] Yasuo S Watanabe M Nakao N Takagi T Follett BK Ebihara S The reciprocal switching of two thyroid hormone-activating and – inactivating enzyme genes is involved in the photoperiodic gonadal response of Japanese quail. Endocrinology (2005) 146:2551–4 10.1210/en.2005-0057 15746251

[22] Bernal J Action of thyroid hormone in brain. J Endocrinol Invest (2002) 25:268–88 11936472 10.1007/BF03344003

[23] Watanabe T Yamamura T Watanabe M Yasuo S Nakao N Dawson A Hypothalamic expression of thyroid hormone-activating and -inactivating enzyme genes in relation to photorefractoriness in birds and mammals. Am J Physiol Regul Integr Comp Physiol (2007) 292:R568–72 10.1152/ajpregu.00521.2006 17197645

[24] Ono H Nakao N Yamamura T Kinoshita K Mizutani M Namikawa T Red jungle fowl (*Gallus gallus*) as a model for studying the molecular mechanism of seasonal reproduction. Anim Sci J (2009) 80:328–32 10.1111/j.1740-0929.2009.00628.x 20163644

[25] Perfito N Jeong SY Silverin B Calisi RM Bentley GE Hau M Anticipating spring: wild populations of great tits *(Parus major*) differ in expression of key genes for photoperiodic time measurement. PLoS One (2012) 7:e34997 10.1371/journal.pone.0034997 22539953 PMC3334499

[26] Stevenson TJ Ball GF Disruption of neuropsin mRNA expression via RNA interference facilitates the photoinduced increase in thyrotropin-stimulating subunit β in birds. Eur J Neurosci (2012) 36:2859–65 10.1111/j.1460-9568.2012.08209.x 22775245

[27] Barrett P Ebling FJ Schuhler S Wilson D Ross AW Warner A Hypothalamic thyroid hormone catabolism acts as a gatekeeper for the seasonal control of body weight and reproduction. Endocrinology (2007) 148:3608–17 10.1210/en.2007-0316 17478556

[28] Freeman DA Teubner BJ Smith CD Prendergast BJ Exogenous T_3_ mimics long day lengths in Siberian hamsters. Am J Physiol Regul Integr Comp Physiol (2007) 292:R2368–72 10.1152/ajpregu.00713.2006 17272662

[29] Herwig A Wilson D Logie TJ Boelen A Morgan PJ Mercer JG Photoperiod and acute energy deficits interact on components of the thyroid hormone system in hypothalamic tanycytes of the Siberian hamster. Am J Physiol Regul Integr Comp Physiol (2009) 296:R1307–15 10.1152/ajpregu.90755.2008 19297543

[30] Watanabe M Yasuo S Watanabe T Yamamura T Nakao N Ebihara S Photoperiodic regulation of type 2 deiodinase gene in Djungarian hamster: possible homologies between avian and mammalian photoperiodic regulation of reproduction. Endocrinology (2004) 145:1546–9 10.1210/en.2003-1593 14726436

[31] Revel FG Saboureau M Pévet P Mikkelsen JD Simonneaux V Melatonin regulates type 2 deiodinase gene expression in the Syrian hamster. Endocrinology (2006) 147:4680–7 10.1210/en.2006-0606 16873538

[32] Yasuo S Yoshimura T Ebihara S Korf HW Temporal dynamics of type 2 deiodinase expression after melatonin injections in Syrian hamsters. Endocrinology (2007) 148:4385–92 10.1210/en.2007-0497 17540726

[33] Ross AW Helfer G Russell L Darras VM Morgan PJ Thyroid hormone signalling genes are regulated by photoperiod in the hypothalamus of F344 rats. PLoS One (2011) 6:e21351 10.1371/journal.pone.0021351 21731713 PMC3120865

[34] Yasuo S Watanabe M Iigo M Nakamura TJ Watanabe T Takagi T Differential response of type 2 deiodinase gene expression to photoperiod between photoperiodic Fischer 344 and nonphotoperiodic Wistar rats. Am J Physiol Regul Integr Comp Physiol (2007) 292:R1315–9 10.1152/ajpregu.00396.2006 17110533

[35] Ono H Hoshino Y Yasuo S Watanabe M Nakane Y Murai A Involvement of thyrotropin in photoperiodic signal transduction in mice. Proc Natl Acad Sci USA (2008) 105:18238–42 10.1073/pnas.0808952105 19015516 PMC2587639

[36] Yasuo S Nakao N Ohkura S Iigo M Hagiwara S Goto A Long-day suppressed expression of type 2 deiodinase gene in the mediobasal hypothalamus of the Saanen goat, a short-day breeder: implication for seasonal window of thyroid hormone action on reproductive neuroendocrine axis. Endocrinology (2006) 147:432–40 10.1210/en.2005-0507 16195409

[37] Hanon EA Lincoln GA Fustin JM Dardente H Masson-Pévet M Morgan PJ Ancestral TSH mechanism signals summer in a photoperiodic mammal. Curr Biol (2008) 18:1147–52 10.1016/j.cub.2008.06.076 18674911

[38] Anderson GM Hardy SL Valent M Billings HJ Connors JM Goodman RL Evidence that thyroid hormones act in the ventromedial preoptic area and the premammillary region of the brain to allow the termination of the breeding season in the ewe. Endocrinology (2003) 144:2892–901 10.1210/en.2003-0322 12810544

[39] Billings HJ Viguié C Karsch FJ Goodman RL Connors JM Anderson GM Temporal requirements of thyroid hormones for seasonal changes in luteinizing hormone secretion. Endocrinology (2002) 143:2618–25 10.1210/en.143.7.2618 12072394

[40] Ikegami K Yoshimura T Circadian clocks and the measurement of daylength in seasonal reproduction. Mol Cell Endocrinol (2012) 349:76–81 10.1016/j.mce.2011.06.040 21767603

[41] Abe T Suzuki T Unno M Tokui T Ito S Thyroid hormone transporters: recent advances. Trends Endocrinol Metab (2002) 13:215–20 10.1016/S1043-2760(02)00599-4 12185668

[42] Hagenbuch B Meier PJ Organic anion transporting polypeptides of the OATP/SLC21 family: phylogenetic classification as OATP/SLCO superfamily, new nomenclature and molecular/functional properties. Pflugers Arch (2004) 447:653–65 10.1007/s00424-003-1168-y 14579113

[43] Nakao N Takagi T Iigo M Tsukamoto T Yasuo S Masuda T Possible involvement of organic anion transporting polypeptide 1c1 in the photoperiodic response of gonads in birds. Endocrinology (2006) 147:1067–73 10.1210/en.2005-1090 16293658

[44] Nicholls TJ Follett BK Robinson JE A photoperiodic response in gonadectomized Japanese quail exposed to a single long day. J Endocrinol (1983) 97:121–6 10.1677/joe.0.0970121 6405006

[45] Nakao N Ono H Yamamura T Anraku T Takagi T Higashi K Thyrotrophin in the pars tuberalis triggers photoperiodic response. Nature (2008) 452:317–22 10.1038/nature06738 18354476

[46] Benoit J Le role des yeux dans l’action stimulante de la lumiere sure le developpement testiulaire chez le canard. C R Soc Biol (Paris) (1935) 118:669–71

[47] Oliver J Bayle JD Brain photoreceptors for the photoinduced testicular response in birds. Experientia (1982) 38:1020–9 10.1007/BF01955346 6751854

[48] Max M McKinnon PJ Seidenman KJ Barrett RK Applebury ML Takahashi JS Pineal opsin: a nonvisual opsin expressed in chick pineal. Science (1995) 267:1502–6 10.1126/science.7878470 7878470

[49] Okano T Yoshizawa T Fukada Y Pinopsin is a chicken pineal photoreceptive molecule. Nature (1994) 372:94–7 10.1038/372094a0 7969427

[50] Siopes TD Wilson WO Extraocular modification of photoreception in intact and pinealectomized coturnix. Poult Sci (1974) 53:2035–41 10.3382/ps.0532035 4462102

[51] Menaker M Roberts R Elliott J Underwood H Extraretinal light perception in the sparrow. III. The eyes do not participate in photoperiodic photoreception. Proc Natl Acad Sci USA (1970) 67:320–5 10.1073/pnas.67.1.320 5272320 PMC283206

[52] Silver R Witkovsky P Horvath P Alones V Barnstable CJ Lehman MN Coexpression of opsin- and VIP-like-immunoreactivity in CSF-contacting neurons of the avian brain. Cell Tissue Res (1988) 253:189–98 10.1007/BF00221754 2970894

[53] Wada Y Okano T Adachi A Ebihara S Fukada Y Identification of rhodopsin in the pigeon deep brain. FEBS Lett (1998) 424:53–6 10.1016/S0014-5793(98)00138-0 9537514

[54] Bailey MJ Cassone VM Melanopsin expression in the chick retina and pineal gland. Brain Res Mol Brain Res (2005) 134:345–8 10.1016/j.molbrainres.2004.11.003 15836930

[55] Chaurasia SS Rollag MD Jiang G Hayes WP Haque R Natesan A Molecular cloning, localization and circadian expression of chicken melanopsin (Opn4): differential regulation of expression in pineal and retinal cell types. J Neurochem (2005) 92:158–70 10.1111/j.1471-4159.2004.02874.x 15606905

[56] Kang SW Leclerc B Kosonsiriluk S Mauro LJ Iwasawa A El Halawani ME Melanopsin expression in dopamine-melatonin neurons of the premammillary nucleus of the hypothalamus and seasonal reproduction in birds. Neuroscience (2010) 170:200–13 10.1016/j.neuroscience.2010.06.082 20620198

[57] Tomonari S Takagi A Akamatsu S Noji S Ohuchi H A non-canonical photopigment, melanopsin, is expressed in the differentiating ganglion, horizontal, and bipolar cells of the chicken retina. Dev Dyn (2005) 234:783–90 10.1002/dvdy.20600 16217736

[58] Tomonari S Takagi A Noji S Ohuchi H Expression pattern of the melanopsin-like (cOpn4m) and VA opsin-like genes in the developing chicken retina and neural tissues. Gene Expr Patterns (2007) 7:746–53 10.1016/j.modgep.2007.06.001 17631423

[59] Davies WI Turton M Peirson SN Follett BK Halford S Garcia-Fernandez JM Vertebrate ancient opsin photopigment spectra and the avian photoperiodic response. Biol Lett (2012) 8:291–4 10.1098/rsbl.2011.0864 22031722 PMC3297396

[60] Halford S Pires SS Turton M Zheng L Gonzalez-Menendez I Davies WL VA opsin-based photoreceptors in the hypothalamus of birds. Curr Biol (2009) 19:1396–402 10.1016/j.cub.2009.06.066 19664923

[61] Nakane Y Ikegami K Ono H Yamamoto N Yoshida S Hirunagi K A mammalian neural tissue opsin (Opsin 5) is a deep brain photoreceptor in birds. Proc Natl Acad Sci USA (2010) 107:15264–8 10.1073/pnas.1006393107 20679218 PMC2930557

[62] Yamashita T Ohuchi H Tomonari S Ikeda K Sakai K Shichida Y Opn5 is a UV-sensitive bistable pigment that couples with Gi subtype of G protein. Proc Natl Acad Sci USA (2010) 107:22084–9 10.1073/pnas.1012498107 21135214 PMC3009823

[63] Groos GA van der Kooy D Functional absence of brain photoreceptors mediating entrainment of circadian rhythms in the adult rat. Experientia (1981) 37:71–2 10.1007/BF01965576 7202675

[64] Legan SJ Karsch FJ Importance of retinal photoreceptors to the photoperiodic control of seasonal breeding in the ewe. Biol Reprod (1983) 29:316–25 10.1095/biolreprod29.2.316 6685536

[65] Lockley SW Skene DJ Thapan K English J Ribeiro D Haimov I Extraocular light exposure does not suppress plasma melatonin in humans. J Clin Endocrinol Metab (1998) 83:3369–72 10.1210/jc.83.9.3369 9745457

[66] Meijer JH Thio B Albus H Schaap J Ruijs ACJ Functional absence of extraocular photoreception in hamster circadian rhythms entrainment. Brain Res (1999) 831:337–9 10.1016/S0006-8993(99)01509-7 10412017

[67] Nelson RJ Zucker I Absence of extraocular photoreception in diurnal and nocturnal rodents exposed to direct sunlight. Comp Biochem Physiol (1981) 69A:145–8 10.1016/0300-9629(81)90651-4

[68] Reiter RJ The pineal and its hormones in the control of reproduction in mammals. Endocr Rev (1980) 1:109–31 10.1210/edrv-1-2-109 6263600

[69] Yamazaki S Goto M Menaker M No evidence for extraocular photoreceptors in the circadian system of the Syrian hamster. J Biol Rhythms (1999) 14:197–201 10.1177/074873099129000605 10452331

[70] Inouye ST Kawamura H Persistence of circadian rhythmicity in a mammalian hypothalamic “island” containing the suprachiasmatic nucleus. Proc Natl Acad Sci USA (1979) 76:5962–6 10.1073/pnas.76.11.5962 293695 PMC411773

[71] Klein DC Moore RY Reppert SM Suprachiasmatic Nucleus: The Mind’s Clock. New York: Oxford University Press (1991).

[72] Lehman MN Silver R Gradstone WR Kahn RM Gibson M Bittman EL Circadian rhythmicity restored by neural transplant. Immunocytochemical characterization of the graft and its integration with the host brain. J Neurosci (1987) 7:1626–38 3598638 10.1523/JNEUROSCI.07-06-01626.1987 PMC6568867

[73] Ralph MR Foster RG Davis FC Menaker M Transplanted suprachiasmatic nucleus determines circadian period. Science (1990) 247:975–8 10.1126/science.2305266 2305266

[74] Arendt J Melatonin and the Mammalian Pineal Gland. London: Chapman & Hall (1995).

[75] Hoffman RA Reiter RJ Pineal gland: influence on gonads of male hamsters. Science (1965) 148:1609–11 10.1126/science.148.3677.1609 14287606

[76] Reppert SM Weaver DR Ebisawa T Cloning and characterization of a mammalian melatonin receptor that mediates reproductive and circadian responses. Neuron (1994) 13:1177–85 10.1016/0896-6273(94)90055-8 7946354

[77] Reppert SM Godson CG Mahle CD Weaver DR Slaugenhaupt SA Gusella JF Molecular characterization of a second melatonin receptor expressed in human retina and brain: the Mel1b-melatonin receptor. Proc Natl Acad Sci USA (1995) 92:8734–8 10.1073/pnas.92.19.8734 7568007 PMC41041

[78] Schuster C Gauer F Guerrero H Lakhdar-Ghazal N Pévet P Masson-Pévet M Photic regulation of mt1 melatonin receptors in the Siberian hamster pars tuberalis and suprachiasmatic nuclei: involvement of the circadian clock and intergeniculate leaflet. J Neuroendocrinol (2000) 12:207–16 10.1046/j.1365-2826.2000.00039.x 10718916

[79] Song CK Bartness TJ CNS sympathetic outflow neurons to white fat that express MEL receptors may mediate seasonal adiposity. Am J Physiol Regul Integr Comp Physiol (2001) 281:R666–72 11448873 10.1152/ajpregu.2001.281.2.R666

[80] Klosen P Bienvenu C Demarteau O Dardente H Guerrero H Pévet P The mt1 melatonin receptor and RORb receptor are co-localized in specific TSH-immunoreactive cells in the pars tuberalis of the rat pituitary. J Histochem Cytochem (2002) 50:1647–57 10.1177/002215540205001209 12486087

[81] Wittkowski W Bergmann M Hoffmann K Pera F Photoperiod-dependent changes in TSH-like immunoreactivity of cells in the hypophysial pars tuberalis of the Djungarian hamster, *Phodopus sungorus*. Cell Tissue Res (1988) 251:183–7 10.1007/BF00215463 3342436

[82] Ebihara S Marks T Hudson DJ Menaker M Genetic control of melatonin synthesis in the pineal gland of the mouse. Science (1986) 231:491–3 10.1126/science.3941912 3941912

[83] Goto M Oshima I Tomita T Ebihara S Melatonin content of the pineal gland in different mouse strains. J Pineal Res (1989) 7:195–204 10.1111/j.1600-079X.1989.tb00667.x 2769571

[84] Yasuo S Yoshimura T Ebihara S Kolf HW Melatonin transmits photoperiodic signals through the MT1 melatonin receptor. J Neurosci (2009) 29:2885–9 10.1523/JNEUROSCI.0145-09.2009 19261884 PMC6666200

[85] Yamamura T Hirunagi K Ebihara S Yoshimura T Seasonal morphological changes in the neuro-glial interaction between gonadotropin-releasing hormone nerve terminals and glial endfeet in Japanese quail. Endocrinology (2004) 145:4264–7 10.1210/en.2004-0366 15178649

[86] Prevot V Croix D Bouret S Dutoit S Tramu G Stefano GB Definitive evidence for the existence of morphological plasticity in the external zone of the median eminence during the rat estrous cycle: implication of neuro-glio-endothelial interactions in gonadotropin-releasing hormone release. Neuroscience (1999) 94:809–19 10.1016/S0306-4522(99)00383-8 10579572

[87] Yamamura T Yasuo S Hirunagi K Ebihara S Yoshimura T T_3_ implantation mimics photoperiodically reduced encasement of nerve terminals by glial processes in the median eminence of Japanese quail. Cell Tissue Res (2006) 324:175–9 10.1007/s00441-005-0126-8 16432711

[88] Jansen HT Cutter C Hardy S Lehman MN Goodman RL Seasonal plasticity within the gonadotropin-releasing hormone (GnRH) system of the ewe: changes in identified GnRH inputs and glial association. Endocrinology (2003) 144:3663–76 10.1210/en.2002-0188 12865349

[89] Yoshimura T Molecular bases for seasonal reproduction in birds. J Poult Sci (2004) 41:251–8 10.1016/j.yfrne.2013.10.002 24157655 PMC3946898

[90] Kasahara T Abe K Mekada K Yoshiki A Kato T Genetic variation of melatonin productivity in laboratory mice under domestication. Proc Natl Acad Sci USA (2010) 107:6412–7 10.1073/pnas.0914399107 20308563 PMC2851971

[91] Shimomura K Lowrey PL Vitaterna MH Buhr ED Kumar V Hanna P Genetic suppression of the circadian clock mutation by the melatonin biosynthesis pathway. Proc Natl Acad Sci USA (2010) 107:8399–403 10.1073/pnas.1004368107 20404168 PMC2889547

[92] Rubin CJ Zody MC Eriksson J Meadows JR Sherwood E Webster MT Whole-genome resequencing reveals loci under selection during chicken domestication. Nature (2010) 464:587–91 10.1038/nature08832 20220755

[93] Nakane Y Ikegami K Iigo M Ono H Takeda K Takahashi D The saccus vasculosus of fish is a sensor of seasonal changes in day length. Nat Commun (2013) 4:2108 10.1038/ncomms3108 23820554

